# MRI Correlates of Disability in African-Americans with Multiple Sclerosis

**DOI:** 10.1371/journal.pone.0043061

**Published:** 2012-08-10

**Authors:** Jonathan Howard, Marco Battaglini, James Scott Babb, Donatello Arienzo, Brigitte Holst, Mirza Omari, Nicola De Stefano, Joseph Herbert, Matilde Inglese

**Affiliations:** 1 Department of Neurology, Langone Medical Center, New York, New York, United States of America; 2 Department of Radiology, Langone Medical Center, New York, New York, United States of America; 3 Department of Neurological and Behavioural Sciences, University of Siena, Siena, Italy; 4 Department of Neuroradiology, University Medical Center Hamburg-Eppendorf, Hamburg, Germany; 5 Department of Neurology Mount Sinai School of Medicine, New York, New York, United States of America; Institute Biomedical Research August Pi Sunyer (IDIBAPS) – Hospital Clinic of Barcelona, Spain

## Abstract

**Objectives:**

Multiple sclerosis (MS) in African-Americans (AAs) is characterized by more rapid disease progression and poorer response to treatment than in Caucasian-Americans (CAs). MRI provides useful and non-invasive tools to investigate the pathological substrate of clinical progression. The aim of our study was to compare MRI measures of brain damage between AAs and CAs with MS.

**Methods:**

Retrospective analysis of 97 AAs and 97 CAs with MS matched for age, gender, disease duration and age at MRI examination.

**Results:**

AA patients had significantly greater T2- (p = 0.001) and T1-weighted (p = 0.0003) lesion volumes compared to CA patients. In contrast, measurements of global and regional brain volume did not significantly differ between the two ethnic groups (p>0.1).

**Conclusions:**

By studying a quite large sample of well demographically and clinically matched CA and AA patients with a homogeneous MRI protocol we showed that higher lesion accumulation, rather than pronounced brain volume decrease might explain the early progress to ambulatory assistance of AAs with MS.

## Introduction

Multiple Sclerosis (MS) is less frequent among African-Americans (AA) than Caucasian-Americans (CA) [1], [2], but it is characterized by a more severe course [3], [4], [5]. MRI provides useful and non-invasive tools to investigate the pathological substrate of clinical progression. Among those, measures of focal white matter (WM) lesions and global brain volumes are well-established, sensitive markers of tissue damage which have shown to be related, to a certain extent, to patient's disability in MS [6]. A previous MRI study comparing MS patients from the two ethnic groups suggested that a higher lesion volume and increased tissue damage as measured by magnetization transfer ratio may explain the rapid clinical progression in AA patients [5]. We hypothesized that in addition to faster lesion accumulation, gray matter (GM) damage may underlie a more aggressive disease course in AA with MS. The purpose of our study was to compare MRI derived selective measures of GM volumes with those of focal WM lesion volumes between subjects with MS of AA and of CA descent matched for age, gender and disease duration.

## Methods

### Patients

The electronic database at the New York University (NYU) Multiple Sclerosis Care Center was searched to identify patients of AA descent. Ethnicity was self-identified by the patient at the time of clinic registration. The diagnosis of MS had to satisfy the International Panel diagnostic criteria [7] and pre-existing medical conditions other than MS had to be excluded. In addition, the patients had to have performed a brain MRI at 1.5 Tesla (from the same manufacturer) at NYU between 2004 an 2009 and had no experienced any relapse with corticosteroid treatment within one month preceding MRI examination. Out of 379 AA patients, 97 fulfilled the clinical and MRI criteria since most patients had had an MRI performed outside NYU on different MRI platforms. The database was then searched alphabetically to identify CA patients who matched the AA subjects based on gender, age, disease duration, age at the time of MRI examination and had a brain MRI performed at 1.5 Tesla at NYU between 2004 and 2009. The search resulted in a total of 97 CA matches. All charts were reviewed to determine the treatment status and exposure, the type of MS (relapsing remitting (RR), secondary progressive (SP), or primary progressive (PP) at the time of MRI and the use of assistive devices for ambulation. The demographic and clinical characteristics of the AA and CA groups are summarized in Table 1. Study approval was obtained from the Institutional Board of Research Associates of NYU Langone Medical Center.

**Table 1:** Demographic and clinical characteristics of the whole group of AA and CA patients and the subgroup with available brain volume measures.

All Patients (n = 194)	AA Patients	CA Patients
Male/Female	19/97 (19.6%)	19/97 (19.6%)
Age at onset (yrs)	33.5±10.6	33.2±10.0
Age at MRI (yrs)	42.7±11.6	42.4±10.8
Disease Duration (yrs)	9.1±7.2	9.5±7.5
Patients under treatment	79/97 (81.4%)	85/97 (85%)
Patients with SP-MS	24/97 (24.7%)	11/97 (11.3%)*
Patients with ambulatory assistance	28/97 (28.9%)	11/97 (11.3%)**

Subset with Volume Data (n = 131)	AA Patients	CA Patients
Male/Female	11/67 (16.4%)	17/64 (26.5%)
Age at onset (yrs)	34.3±10.8	33.9±10.1
Age at MRI (yrs)	43.9±11.7	43.8±10.7
Disease Duration (yrs)	9.6±6.8	10.3±8.4
Patients under treatment	60/67 (89.6%)	55/64 (85.9%)
Patients with SP-MS	15/67 (22.4%)	9/64 (14.1%)
Patients with ambulatory assistance	19/67 (28.4%)	8/64 (12.5%)**

* p = 0.04 (Fisher exact test); **p<0.01 (binary logistic regression analysis adjusted for age, gender, disease duration treatment type and exposure).

**Table 2:** Lesion and brain volume measures (mean ± SD) in the whole group of AA and CA patients and in the subgroup with available brain volume measures.

All Patients (n = 194)	AA patients	CA patients	p value
CELs (n)	0.64±2.62	0.13±0.51	0.4
T2 LV (mL)	13.40±17.61	7.04±9.01	**0.001**
T1 LV (mL)	1.31±2.23	0.53±0.97	**0.0003**
T1/T2 LV	0.10±0.10	0.10±0.19	0.07

Subset with volume data (n = 131)	AA patients	CA patients	
CELs (n)	0.34±1.15	0.16±0.50	0.7
T2 LV (mL)	13.57±16.22	7.88±10.23	**0.006**
T1 LV (mL)	1.39±2.45	0.64±1.16	**0.002**
T1/T2 LV	0.10±0.09	0.11±0.18	0.05
NBV (mL)	1557.82±113.93	1558.54±130.58	0.8
NWMV (mL)	730.47±65.39	739.00±66.05	0.5
NGMV (mL)	921.72±76.20	819.55±86.74	0.4

CELs  =  contrast-enhancing lesions; LV  =  lesion volume; NBV  =  normalized brain volume; NWMV  =  normalized white matter volume; NGMV  =  normalized gray matter volume.

**Table 3:** Lesion and brain volume measures in whole MS patients stratified by ambulatory status, in CA MS and in AA patients stratified byambulatory status.

MRI	All patients	All patients	p value	CA patients	AA patients	p value	CA patients	AA patients	p value
	w/o aid	with aid		w/o aid	w/o aid		with aid	with aid	
CELs	0.21±0.80	1.10±3.89	0.08	0.14±0.53	0.29±1.04	0.6	0.09±0.30	1.50±4.54	0.3
T2 LV	7.71±10.29	20.19±21.99	**<0.0001**	6.29±8.45	9.49±12.03	**0.03**	12.89±11.35	23.06±24.54	0.4
T1 LV	0.66±1.16	1.95±2.98	**<0.0001**	0.50±0.96	0.86±1.35	**0.02**	0.79±1.10	2.40±3.37	**0.02**
T1/T2 LV	0.10±0.16	0.11±0.09	0.05	0.11±0.20	0.09±0.10	0.2	0.07±0.06	0.12±0.09	0.1
NBV	1577.16±111.52	1485.05±134.18	**0.001**	1575.2±118.5	1579.45±103.98	0.8	1441.97±159.35	1503.19±122.31	0.3
NWMV	741.05±63.60	709.91±68.48	**0.02**	745.45±62.45	735.93±65.20	0.5	693.86±77.15	716.67±65.55	0.5
NGMV	896.90±61.82	775.13±90.08	**0.0008**	829.75±78.80	975.24±89.72	0.1	748.10±99.70	786.52±80.58	0.4

CELs  =  contrast-enhancing lesions (number); LV  =  lesion volume (mL); NBV  =  normalized brain volume (mL); NWMV  =  normalized white matter volume (mL); NGMV  =  normalized gray matter volume. (mL).

### MRI Study

All MRIs were performed at NYU Medical Center on a Siemens 1.5T MRI scanner (Magnetom Vision, Sonata, Symphony or Avanto, Siemens Medical Solutions, Erlangen, Germany). The clinical MRI protocol included axial T2-weighted turbo spin-echo (TSE), sagittal fluid attenuated inversion recovery (FLAIR), 3D Magnetization Prepared Rapid Acquisition Gradient-echo (MPRAGE) and T1-weighted spin-echo (SE) with and without Gadolinium (Gd) contrast. The *axial TSE T2* sequence was acquired with TR ranging from 5400 to 4000 ms, TE ranging from 104 to 99 ms, FOV 220×220 mm^2^, matrix 256×256, with a total of 22–24 5-mm-thick slices. We also acquired sagittal *3D-SPGR T1-WI* scans with TR ranging from 9700 to 2100 ms, TE ranging from 4 to 3.67 ms and TI ranging from 3000 to 1100 ms, FOV 220×220 mm^2^, matrix 256×256, with 170–185, 1.0-mm-thick, slices, no gap; *sagittal FLAIR* with TR 8002 ms, TE 128 ms, TI 2000 ms, FOV 220×220 mm^2^, matrix 192×256, 22–24 5-mm thick slices; *axial SE T1-WI* was acquired with TR ranging from 613 to 366 ms, TE ranging from 11 to 17 ms, FOV 220×220 mm^2^, matrix 256×256, 22–24 5-mm-thick slices. The Gd-enhanced SE T1-WI sequence was obtained after injection of a single dose intravenous bolus (0.1 mMol/Kg Gd-DTPA) 5 min after administration of contrast agent. Lesion volume analysis was performed on 97 AA and 97 CA MS patients. Brain volume analysis was performed on 67 AA and 64 CA MS patients due to either lack or low quality of MPRAGE images. The subgroups of AA and CA MS patients whose images qualified for the brain volume analysis did not differ in terms of demographical and clinical characteristics from the original cohort (Table 1).

### Lesion count and volume assessment

For all subjects, Gadolinium contrast-enhancing (CE) lesion number, T2-hyperintense and T1-hypointense lesion volume (LV) measurements were performed by a single experienced observer, blinded to the subject's identity, race and clinical characteristics using a semiautomated segmentation technique based on user-supervised local thresholding as described elsewhere [8].

### Brain volume assessment

For the 67 AA and 64 CA MS patients with available MPRAGE images, normalized brain volume (NBV), gray, and white matter volumes (NGMV and NWMV) were measured by two experienced observers blinded to the subject's identity, race and clinical characteristics using SIENAX [9]. The analysis was corrected for the impact of WM lesions on brain measurements [9].

### Statistical Methods

A Fisher exact test was used to compare AAs and CAs with respect to gender, treatment type and MS type. An exact Mann-Whitney test was used to compare AAs and CAs with respect to age at disease onset, age at time of MRI, disease duration and treatment exposure. A binary logistic regression was used to compare AAs and CAs with respect to ambulatory status correcting for age, gender, disease duration, treatment type and exposure. For this analysis, ambulatory status was the dependent variable and the logistic model included subject group (CA vs. AA) and age, gender and disease duration as covariates. Analysis of covariance (ANCOVA) based on ranks was used to compare AA and CA with respect to MRI measures correcting for gender, age at onset, disease duration, MS type, treatment and treatment exposure. A separate ANCOVA was conducted for each MR measure using the ranks of the measure as the dependent variable. Each ANCOVA model included subject group as a classification factor and gender, age, disease duration, MS type, treatment and treatment exposure as subject-level covariates. ANCOVA based on ranks was also used to compare patients with different ambulatory status in terms of the MR measures adjusted for the covariates; these comparisons were conducted using data from all patients and then separately for CA and AA. All reported p values are two-sided with statistical significance defined as p<0.05. SAS 9.0 (SAS Institute, Cary, NC) was used for all statistical computations.

## Results

AA and CA patients were not significantly different in terms of age at disease onset, age at MRI and disease duration at time of MRI (Table 1). Additionally, there was no significant difference between the two ethnic groups in terms of treatment exposure at the time of MRI (6.07±4.3 yrs for AAs and 6.4±4. 4 yrs for CAs patients; p = 0.4). The proportion of patients on disease modifying therapies was 81.4% for the AA group and 85% for the CA group. 49.5% of AA and 54.7% of CA were on interferon-β therapy (AA: 14/48 =  beta 1a Rebif®; 29/48 =  beta 1a Avonex®; 5/48 =  beta 1b Betaseron®; CA: 7/53 =  Rebif®; 40/53 =  Avonex®; 6/53 =  Betaseron®), 10.3% AA and 12.4% CA were on glatiramer acetate, 10.3% AA and 8.3% CA were on natalizumab and 11.4% AA and 6.2% CA were on immunosuppressive therapies including azathioprine, mitoxantrone and intravenous immunoglobulin. AA patients in our series had a more severe disease course compared to CA patients, with 28.9% requiring ambulatory assistance compared to 11.3% of CA patients (p value  = 0.0013). There were 85.6% (83/97) patients with RR-MS, 3.1% (3/97) with PP-MS and 11.3% (11/97) with SP-MS in the CA group and 71.1% (69/97) patients with RR-MS, 4.1% (4/97) with PP-MS and 24.7% (24/97) with SP-MS in the AA group. AA patients had significantly greater T2- and T1-LV but similar number of contrast-enhancing lesions compared to CA patients in both the groups of patients as a whole and in the subgroup of patients whose images qualified for brain volume measures (Table 2). The proportion of AA patients with contrast-enhancing lesions was 13.4% (13/97) whereas the proportion of CA patients was 8.2% (8/97). Measurements of NBV, NGMV and NWMV did not significantly differ between the two ethnic groups (Table 2). However, when all patients regardless of ethnicity were stratified based on the ambulatory status, significant differences in NBV, GMV, WMV, T1LV and T2LV were found between the group of patients who did not require ambulatory aid and the group who needed ambulatory aid (Table 3). In contrast, when subjects were subdivided based on ethnicity and ambulatory status, only T2- and T1-LVs were significantly higher in AA than CA patients (Table 3).

## Discussion

The biological correlates of a more rapidly disabling disease course in AA patients are not clear [10], [11]. A recent MRI study comparing patients from the two ethnic groups has shown that AA patients with MS suffered more severe diffuse tissue damage, as measured by magnetization transfer ratio, and higher lesion volumes compared to CA patients [5]. Our study adds to previous work by clearly showing higher T2- and T1-LV in the more disabled AA MS group than in the CA MS patients, further supporting the role of lesion accumulation and lesion evolution into black holes as correlates of the more aggressive clinical course in AA MS patients.

We did not find here any significant difference in global and regional brain volume between the two ethnic groups. This is somehow surprising considering the significant higher lesion burden in AA patients and might be explained by ethnic differences in brain volumes between AA and CA subjects [12]. Admittedly, since the high-resolution 3D T1-weighted sequence was not available for the entire cohort of patients, the volumetric analysis was performed in a subgroup of 67 AA and 64 CA patients (see Table 1 and Table 2). Therefore, the lack of any difference in terms of brain atrophy could be due to the low number of patients analyzed. It is also possible, that our volumetric approach including global measures of whole brain, and WM and GM atrophy is not sensitive enough to detect regional differences in WM and GM volume between the two groups. However, when all patients, regardless of ethnic background, were stratified according to clinical status, those who required ambulatory assistance had significant lower NBV, NGMV and NWMV than those who did not require any assistance. This is consistent with the notion that not only lesion accumulation but also brain tissue loss contributes to development of irreversible disability [6] Prospective longitudinal studies with large samples will be needed to track the development of atrophy and its clinical impact in AA patients with MS.

Limitations of our study lie in its retrospective design, in the assessment of disability on the requirement of ambulatory assistance without the inclusion of more sophisticated measures of physical and cognitive decline and, finally, in a routine MRI protocol, which did not include modern sequences for the evaluation of cortical lesions and damage in the normal-appearing tissue. Nonetheless, by studying a quite large sample of well demographically and clinically matched CA and AA patients with a homogeneous MRI protocol we were able to provide further insights into the mechanisms of disease progression in AA patients with MS.
