# Correction: MRI Correlates of Disability in African-Americans with Multiple Sclerosis

**DOI:** 10.1371/annotation/25df480c-60b5-43a3-b03c-4e97d6ee399c

**Published:** 2013-06-07

**Authors:** Jonathan Howard, Marco Battaglini, James Scott Babb, Donatello Arienzo, Brigitte Holst, Mirza Omari, Nicola De Stefano, Joseph Herbert, Matilde Inglese

Author Mirza Omari was incorrectly mentioned in the Funding Statement. The following is the correct Funding Statement:

The study was funded in part by National institute of health (NIH) [grant R01 NS051623 to Matilde Inglese]. No additional external funding received for this study. The funders had no role in study design, data collection and analysis, decision to publish, or preparation of the manuscript.

Author Mirza Omari was incorrectly mentioned in the Competing Interest Statement. The following is the correct Competing Interest Statement:

Nicola De Stefano serves on the scientific advisory boards for Merck Serono and Novartis; he has received funding for travel from Merck Serono, Novartis and Teva Pharmaceutical Industries ltd.; he has received speaker honoraria from Novartis, Teva Pharmaceutical lndustries ltd., Biogen-Dompé, Schering-Bayer, Sanofi-Aventis, and Merck Serono. Joseph Herbert has received grants from Biogen, Teva, Merk Serono and Bayer. Matilde Inglese is funded by NIH grant R01 NS051623. Matilde Inglese serves on the scientific advisory board of Celgene and she has served as consultant for Vaccinex and Biogen. This does not alter the authors’ adherence to all the PLoS ONE policies on sharing data and materials. 

